# Modified artificial bee colony for the vehicle routing problems with time windows

**DOI:** 10.1186/s40064-016-2940-8

**Published:** 2016-08-09

**Authors:** Malek Alzaqebah, Salwani Abdullah, Sana Jawarneh

**Affiliations:** 1 Department of Computer Science, Jadara University, Irbid, Jordan; 2 Data Mining and Optimisation Research Group, Centre for Artificial Intelligence Technology, Universiti Kebangsaan Malaysia, 43600 Bangi, Selangor Malaysia

**Keywords:** Foraging behaviour, Artificial bee colony, Vehicle routing problem with time windows

## Abstract

The natural behaviour of the honeybee has attracted the attention of researchers in recent years and several algorithms have been developed that mimic swarm behaviour to solve optimisation problems. This paper introduces an artificial bee colony (ABC) algorithm for the vehicle routing problem with time windows (VRPTW). A Modified ABC algorithm is proposed to improve the solution quality of the original ABC. The high exploration ability of the ABC slows-down its convergence speed, which may due to the mechanism used by scout bees in replacing abandoned (unimproved) solutions with new ones. In the Modified ABC a list of abandoned solutions is used by the scout bees to memorise the abandoned solutions, then the scout bees select a solution from the list based on roulette wheel selection and replace by a new solution with random routs selected from the best solution. The performance of the Modified ABC is evaluated on Solomon benchmark datasets and compared with the original ABC. The computational results demonstrate that the Modified ABC outperforms the original ABC also produce good solutions when compared with the best-known results in the literature. Computational investigations show that the proposed algorithm is a good and promising approach for the VRPTW.

## Background

Many real transportation logistics and distribution problems can be expressed as a vehicle routing problem, where the objective is to plan a route with minimum cost while serving a set of customers with known demands. Each customer is allocated to only one route and the total demand of any route must not exceed the vehicle capacity.

The vehicle routing problem with time windows (VRPTW) is a well-known optimisation problem and it has received a lot of consideration in the literature. The VRPTW involves determining a set of routes starting and ending at a depot, wherein the demand of a set of geographically scattered customers is satisfied. Each route is traversed by a vehicle with a permanent and limited capacity, and each customer needs to be visited only once. The total demand (load) delivered in each route should not exceed the vehicle’s capacity. Time windows are imposed for the customer destinations, meaning that the vehicle is only permitted to arrive at/depart from a customer destination within a specific time window. The solution to the VRPTW consists of the set of routes that has the least total travelled distance (Desrochers et al. 1988a, b). Surveys papers in VRPTW can be founded in Kumar and Panneerselvam (2012), Bräysy and Gendreau (2005a, b), Ioannou et al. (2001), Toth and Vigo (2001), Toth and Vigo (2014) and Solomon and Desrosiers (1988).

Observations on the swarm behaviour of natural self-organised systems has attracted researchers’ attention in recent years and inspired the development of algorithms for solving real-life problems; these algorithms are known as swarm intelligence. Examples of swarm intelligence that have been applied to the VRPTW are bee colony optimisation (Jawarneh and Abdullah 2015), ant colony optimisation (Gambardella et al. 1999; Yu et al. 2009) and particle swarm optimisation (Amini et al. 2010). A swarm intelligence algorithm simulates the behaviour of the self-organised system. In particular, the behaviour of real honeybees has motivated researchers to develop metaheuristics that model such behaviour in order to find better solutions for optimisation problems. Honeybee algorithms are classified into three different types based on behaviour (Baykasoglu et al. 2007): foraging, marriage and queen bee. The algorithms that are based on the foraging behaviour of honeybees to solve optimisation problems are the artificial bee colony (ABC), bee algorithm (BA) and bee colony optimisation (BCO). The bees in these three algorithms have different behaviour models. In this paper, the focus is on the ABC algorithm.

The ABC algorithm was proposed Karaboga (2005) for solving numerical optimisation problems. A further updated version of the ABC algorithm was later proposed by Karaboga and Basturk (2008) for solving constraint optimisation problems, Szeto et al. (2011) introduced an artificial bee colony heuristic for solving the capacitated vehicle routing problem (CVRP) and they show that the enhanced ABC algorithm outperforms the original one, and can produce good solutions when compared with other heuristics. The ABC algorithm works based on local communication between three groups of bees (scouts, employed and onlookers) and between each other about their environment, which contributes to the collective intelligence of the bee colony (Karaboga 2005).

In this paper, a new approach is proposed to improve the convergence speed of the ABC algorithm by introducing a method of finding abandoned solutions and replacing them with new solution that has a random routs selected from the best solution found so far. Experimental results show that the proposed Modified ABC algorithm improves the results. Overall comparison indicates that our Modified ABC algorithm is able to obtain the best results in comparison to state-of-the-art approaches, as represented by reducing the distance travelled, which is the main objective of the VRPTW.

This paper starts with representation of the VRPTW and its formulation. Next, introduction of the ABC algorithm and the modified ABC algorithm are presented. At last, the experimental results are presented and analysed.

## Vehicle routing problems with time windows (VRPTW)

The VRPTW consists of a set of identical vehicles, a depot node, a limited number of distributed customers, and a net connecting all the customers to the depot. Solomon (1987) proposes the formulation of the VRPTW, Solomon’s benchmark has 56 instances with 100 customers must be served by a predefined number of vehicles. Each arc in the network shows a connection between two nodes and identifies the direction of travel. Every vehicle starts from the depot, arrives at some customer destinations, and then returns to the depot. Every vehicle illustrates one route in the network. In Solomon’s benchmark, the cost *c*_*ij*_ and the travel time *t*_*ij*_ are linked with every arc in the network; the vehicle spends one unit of time to travel one unit of distance (Euclidean distance).

Every vehicle has the same capacity and each customer must be visited one time only by one of the vehicles and has an identified demand, and the total capacity of all the demands must be equal or less than the maximum capacity of the vehicle on the route being travelled, and no vehicles should be overloaded. The time window constraints are indicated by a predefined time interval, i.e., the earliest arrival time and latest arrival time, where the vehicle must arrive at the customer before the latest arrival time and if it arrives before the earliest arrival time, the vehicle must wait. The service time for each customer for the time taken to load/unload is also measured, which is considered to be unique regardless of the demands’ size. Vehicles are considered to visit their route within route time, which is named as the time window.

The main function in the problem is illustrated in Eq. 1:

1 $${\text{Minimise}} \mathop \sum \limits_{i = 0}^{N} \mathop \sum \limits_{j = 0,j \ne i}^{N} \mathop \sum \limits_{k = 1}^{K} c_{ij } x_{ijk}$$

where

- $$x_{ijk} = \left\{ {\begin{array}{*{20}c} 0 & {{\text{if there is no arc from node}} {\text{i to node j}}} \\ 1 & {\text{otherwise }} \\ \end{array} i \ne j, i, j \in \{ 0,1,2, \ldots N\} } \right.$$.
- *N number of customers (0 denotes the central depot)*
- *K number of vehicles*
- *c*_*ij*_*cost incurred on arc from customer i to customer j.*

The following constraints represent the time windows without violating any time window constraints, where for each vehicle, the earliest possible time of returning to the depot cannot exceed the latest possible return time.

2 $$\mathop \sum \limits_{k = 1}^{K} \mathop \sum \limits_{i = 0,j \ne i}^{N} x_{ijk} \left( {t_{i} + t_{ij} + f_{i} + w_{i} } \right) \le t_{j } {\text{for}} j \in \left\{ {1,2, \ldots ,N} \right\}$$

3 $$e_{i} \le (t_{i} + w_{i} ) \le l_{j }\quad for\quad i \in \left\{ {1,2, \ldots ,N} \right\}$$

## Artificial bee colony algorithm (ABC)

The ABC algorithm simulates the foraging behaviour of real honeybees. Several researchers have studied its application to optimisation problems in recent years. A survey paper on algorithms based on honeybee behaviour (ABC, bee colony algorithm, bee swarm optimisation, bees algorithm, and honeybee mating optimisation) by Karaboga et al. (2014) reported that 54 % of recent publications are related to the ABC algorithm.

In the ABC algorithm, there are three groups of agents (employed bees, onlooker bees and scout bees) that work collaboratively in solving problems. The groups of agents communicate and cooperate to find a food source (solution) with good-quality nectar (cost or fitness value for a problem). Each group of agents is responsible for one process. The scout bees are responsible for finding the positions of new food sources. At the beginning of the search, the food source positions are determined randomly. The employed bees are responsible for exploring the search space and visit the neighbourhood of the food source positions collecting all the information about the visited positions (including the distance, direction and profitability) in order to share this information with the onlooker bees. The onlooker bees are responsible for selecting promising food sources based on the information given by the employed bees.

In the ABC algorithm, a food source represents a possible solution, and the amount of nectar in the food source corresponds to the quality (the cost or the fitness value) of the solution. The number of employed bees is equal to the number of solutions in the population.

**Fig. 1:**
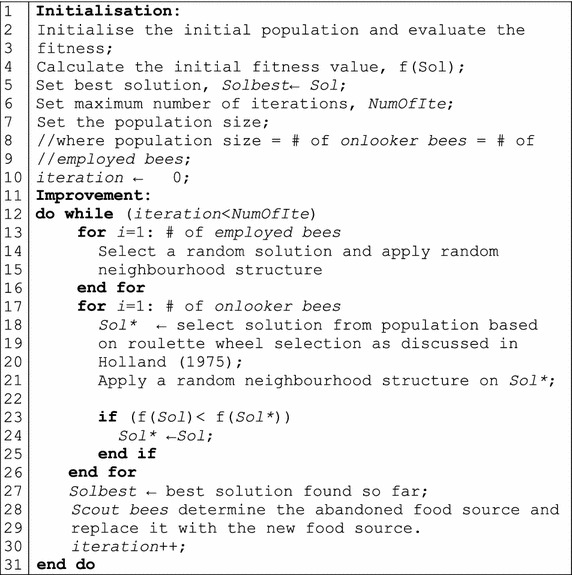
Pseudo code for ABC algorithm

As shown in Fig. 1, the ABC algorithm starts by initialising the population with respect to the number of employed bees. Employed bees explore new solutions based on their memories and old solutions are replaced by new explored solutions only if the new solutions have an equal or better fitness value.

Communication between the employed and onlooker bees happens when the employed bees present promising solutions to the onlooker bees in the hive. Cooperation happens when the onlooker bees explore the solutions presented by the employed bees. If the solution cannot be improved in a predefined number of iterations (defined as a parameter called *limit*), the employed bee becomes a scout bee in order to explore a new solution. At this point, the newly discovered solution is memorised and the old solution is abandoned. The ABC algorithm has exploration and exploitation capabilities, where scout bees are able to globally explore the search space and employed and onlooker bees are able to locally explore the search space.

## Modified ABC algorithm

The basic ABC algorithm described above still lacks the ability to execute strong exploration throughout the scout bee phase. The main weakness of the basic ABC algorithm is that scout bees promote high exploration capability because a new solution is created randomly when the old solution cannot be improved. Randomly created solutions may affect the search process to search the area blindly making it difficult to find the promising areas.

This causes the search to slow the convergence speed (Weng and Bin Asmuni 2013). Therefore, in this paper we propose an improved version of the ABC algorithm, where a list of abandoned solutions is defined (abnd_lst: extendable abandoned list) so that the scout bees can memorise the solutions that exceed the maximum number of trials (*limit*).

**Fig. 2:**
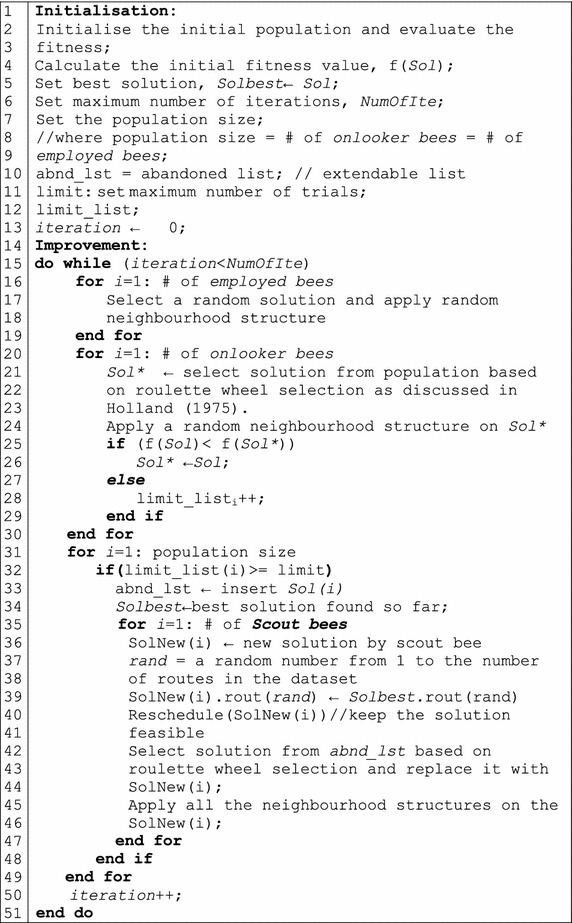
Pseudo code for Modified ABC algorithm

The scout bees select a solution from the abandoned list based on a roulette wheel selection. Then each scout bee creates a new solution by selecting a random route from the best solution found so far, and replace it with a random route in the new generated solution (Fig. 2, line 39), while keeping the solution feasible (Fig. 2, line 40), and all the neighbourhood structures are performed on each new created solution in order to slightly improve the new generated solutions. The pseudo code for the Modified ABC algorithm is presented in Fig. 2.

## Neighbourhood structures

To explain the neighbourhood structures, let’s consider that *a*, *b*, *c* and *d* are customers in the same route visited in sequence {*a* → *b* → *c* → *d*}. The neighbourhood structures presented as follows:

NBS1: One shift in the same route, where *b* is moved to a position after all the other customers’ positions; the new sequence is {*a* → *c* → *d* → *b*}
NBS2: Two shifts in the same route, where both *a* and *b* are moved to a position after *c* and *d*; the new sequence is {*c* → *d* → *a* → *b*}
NBS3: One swap in the same route, where *a* and *c* are exchanged; the new sequence is {c → b → *a* → *d*}
NBS4: Two swaps in the same route, where a and b are exchanged with c and d, the new sequence is {c → d → a → b}

## Solomon’s benchmark for VRPTW

Solomon in 1987 proposes 6 datasets for the VRPTW, which have been used by a number of heuristics. In this paper, these benchmark datasets are used to test the performance of the Modified ABC algorithm. The instances vary in terms of the number of vehicles used to serve the customers, travelling time of the vehicles, vehicle capacity, customer locations and service time (for unloading/loading). In other words, each customer has their own time window, demand (amount of properties), location, earliest arrival time and latest arrival time, and service time.

All instances in the benchmark datasets have 100 customers; the travelling time among customers is equivalent to the same Euclidean distance. The 56 instances are splitted into six groups based on the arrangement of the customers’ locations and time windows. These groups are C1, C2, R1, R2, RC1, and RC2. In group C the customers are clustered, while in group R the customers are distributed remotely. Group RC mixes the customers distribution in the instances R and C.

## Results

The experiment results are based on 31 independent runs. Our proposed algorithms were implemented in Java programming language on Intel^®^ Core™ i3 processors. The execution times were between 20 and 700 s based on the size of the tested dataset.

Table 1 shows the final parameter settings, which were experimentally chosen from a total of 10 runs to obtain the average results.

**Table 1:** Final parameter settings

Parameter	Value
# of iterations	1000
Population size = # of onlooker bees = # of employed bees	50
# of scout bees	4
Limit	100

 Table 2 shows the comparison of the results of the ABC and Modified ABC algorithms for VRPTW.

**Table 2:** Comparison of ABC and Modified ABC

No	Instance	ABC			Modified ABC			*p* value
		N.V	Distance		N.V	Distance		
			Best	Avg		Best	Avg	
0	R1-01	20	1647.99	1660.87	20	1643.18	1647.91	0.1450
1	R1-02	18	1498.67	1505.03	18	1480.73	1490.66	0.0917
2	R1-03	15	1280.51	1287.58	14	1240.87	1258.50	*0.0188*
3	R1-04	12	1085.33	1100.40	12	1047.06	1070.27	0.0648
4	R1-05	16	1404.43	1419.40	16	1369.52	1382.35	*0.0228*
5	R1-06	14	1320.96	1325.17	13	1271.13	1285.81	*0.0038*
6	R1-07	13	1162.02	1176.49	12	1129.99	1142.23	0.1057
7	R1-08	12	1067.73	1073.98	11	1004.11	1026.11	*0.0070*
8	R1-09	14	1248.79	1258.29	13	1170.50	1211.12	0.0850
9	R1-10	13	1191.85	1197.11	12	1123.36	1145.30	*0.0027*
10	R1-11	13	1158.56	1168.90	12	1101.59	1129.55	*0.0384*
11	R1-12	12	1075.84	1087.28	11	1019.84	1026.25	*0.0111*
12	R2-01	10	1222.49	1232.49	8	1185.57	1192.87	*0.0264*
13	R2-02	9	1148.33	1162.38	7	1103.15	1114.87	*0.0085*
14	R2-03	7	1025.26	1033.58	6	958.94	984.34	*0.0319*
15	R2-04	5	894.45	908.58	4	818.44	836.49	*0.0020*
16	R2-05	7	1066.51	1077.50	6	1020.53	1032.79	*0.0099*
17	R2-06	6	1009.90	1016.53	5	960.29	976.45	*0.0353*
18	R2-07	6	945.16	968.24	5	905.70	930.46	0.0592
19	R2-08	4	869.56	878.96	4	764.90	789.02	*0.0015*
20	R2-09	6	972.40	990.24	6	943.16	952.73	*0.0478*
21	R2-10	7	1039.74	1048.75	6	1003.91	1015.11	*0.0238*
22	R2-11	5	914.13	916.70	5	837.66	855.79	*0.0035*
23	C1-01	10	828.94	903.63	10	828.94	828.94	0.1835
24	C1-02	11	917.39	958.27	10	828.94	828.94	0.1835
25	C1-03	10	867.44	886.63	10	828.94	840.66	0.0750
26	C1-04	10	923.46	948.36	10	858.90	889.10	*0.0213*
27	C1-05	11	868.14	915.03	10	828.94	828.94	0.1835
28	C1-06	10	830.46	910.91	10	828.94	828.94	0.1835
29	C1-07	11	862.82	923.86	10	828.94	828.94	0.1835
30	C1-08	10	839.94	840.39	10	828.94	830.85	*0.0003*
31	C1-09	10	855.37	862.07	10	828.94	836.47	*0.0398*
32	C2-01	3	591.56	591.56	3	591.56	591.56	0.1835
33	C2-02	3	591.56	623.18	3	591.56	601.78	0.1835
34	C2-03	4	670.82	686.86	3	600.54	616.39	*0.0094*
35	C2-04	4	761.83	779.50	3	610.01	648.57	*0.0028*
36	C2-05	3	589.72	606.48	3	588.88	596.10	*0.0004*
37	C2-06	3	600	621.78	3	588.88	601.49	*0.0000*
38	C2-07	3	610.64	638.27	3	589.58	601.60	0.1038
39	C2-08	3	625.96	648.24	3	591.65	613.47	*0.0476*
40	RC1-01	17	1701.49	1707.32	16	1634.52	1668.07	0.0854
41	RC1-02	15	1538.02	1554.90	15	1492.89	1505.94	*0.0147*
42	RC1-03	13	1406.80	1410.79	13	1334.57	1360.15	*0.0137*
43	RC1-04	12	1295.99	1300.80	11	1215.62	1245.35	*0.0281*
44	RC1-05	17	1641.95	1650.60	15	1546.43	1575.46	*0.0048*
45	RC1-06	14	1487.54	1499.55	14	1423.10	1443.77	*0.0108*
46	RC1-07	13	1353.65	1369.73	12	1300.00	1324.00	0.0660
47	RC1-08	12	1300.08	1304.05	12	1193.68	1213.67	*0.0147*
48	RC2-01	9	1374.51	1376.78	8	1308.76	1320.24	*0.0079*
49	RC2-02	9	1238.70	1246.74	8	1167.00	1180.48	*0.0041*
50	RC2-03	6	1096.83	1096.85	6	1014.79	1032.77	*0.0052*
51	RC2-04	5	940.85	956.02	4	881.88	894.76	*0.0319*
52	RC2-05	9	1270.79	1289.07	7	1210.68	1232.84	*0.0413*
53	RC2-06	8	1184.85	1199.93	6	1112.38	1133.99	*0.0214*
54	RC2-07	7	1126.12	1137.30	7	1059.62	1076.47	*0.0041*
55	RC2-08	5	937.11	958.45	5	882.06	898.45	0.0884

*NV* number of vehicles

To compare the performance of the algorithms a single objective is used, the distance is considered as the main objective. The comparison in Table 2 shows that the Modified ABC algorithm outperforms the ABC algorithm in all the tested datasets; the new method for scout bees further enhances the quality of the solutions. A statistical test is executed to examine if there is any significant difference between the ABC and the Modified ABC algorithms with the significance interval 95 % (α = 0.05). The *p* value shows that there are significant differences in 38 datasets, which means that 67 % of the results are significant. Note that the italic font means that the *p*-value is less than 0.05.

Table 3 shows the comparison results between the Modified ABC algorithm and the best-known results in the literature. Note that the best results (distances) are presented in bold and the last column shows the deference percentage between the Modified ABC and the best known results, where the minus sign indicate that the modified ABC obtain better results.

**Table 3:** Comparison between the best-known results and Modified ABC

No	Instance	Best-known result		Source	Modified ABC		Percentage difference between Modified ABC with the best known solutions (%)
		N.V	Distance		N.V	Distance	
0	R1-01	18	*1607.70*	Desrochers et al. (1992)	20	1643.18	2.21
1	R1-02	17	*1434.00*	Desrochers et al. (1992)	18	1480.73	3.26
2	R1-03	13	*1175.67*	Lau et al. (2001)	14	1240.87	5.55
3	R1-04	10	*982.01*	Rochat and Tailard (1995)	12	1047.06	6.62
4	R1-05	15	*1346.12*	Kallehauge et al. (2001)	16	1369.52	1.74
5	R1-06	13	*1234.6*	Cook and Rich (1999)	13	1271.13	2.96
6	R1-07	11	*1051.84*	Kallehauge et al. (2001)	12	1129.99	7.43
7	R1-08	9	*960.88*	Berger and Barkaoui (2004)	11	1004.11	4.50
8	R1-09	12	*1013.2*	Chiang and Russel (1997)	13	1170.5	15.53
9	R1-10	12	*1068.00*	Cook and Rich (1999)	12	1123.36	5.18
10	R1-11	12	*1048.70*	Cook and Rich (1999)	12	1101.59	5.04
11	R1-12	10	*953.63*	Rochat and Tailard (1995)	11	1019.84	6.94
12	R2-01	4	1252.37	Homberger and Gehring (1999)	8	*1185.57*	−5.33
13	R2-02	3	1158.98	Lau et al. (2003)	7	*1103.15*	−4.82
14	R2-03	3	*939.50*	Woch and Lebkowski (2009)	6	958.94	2.07
15	R2-04	2	825.52	Bent and Van Hentenryck (2004)	4	*818.44*	−0.86
16	R2-05	3	994.42	Rousseau et al. (2002)	6	1020.53	2.63
17	R2-06	3	*833.00*	Thangiah et al. (1994)	5	960.29	15.28
18	R2-07	3	*814.78*	Rochat and Tailard (1995)	5	905.7	11.16
19	R2-08	2	*726.75*	Mester et al. (2007)	4	764.9	5.25
20	R2-09	3	*855.00*	Thangiah et al. (1994)	6	943.16	10.31
21	R2-10	3	*939.34*	Mester et al. (2007)	6	1003.91	6.87
22	R2-11	2	877.55	Yu et al. (2009)	5	*837.66*	−4.55
23	C1-01	10	*827.30*	Desrochers et al. (1992)	10	828.94	0.20
24	C1-02	10	*827.30*	Desrochers et al. (1992)	10	828.94	0.20
25	C1-03	10	*826.30*	Tavares et al. (2003)	10	828.94	0.32
26	C1-04	10	*822.90*	Tavares et al. (2003)	10	858.9	4.37
27	C1-05	10	*827.30*	Tavares et al. (2003)	10	828.94	0.20
28	C1-06	10	*827.30*	Desrochers et al. (1992)	10	828.94	0.20
29	C1-07	10	*827.30*	Tavares et al. (2003)	10	828.94	0.20
30	C1-08	10	*827.30*	Tavares et al. (2003)	10	828.94	0.20
31	C1-09	10	*827.30*	Tavares et al. (2003)	10	828.94	0.20
32	C2-01	3	*589.10*	Cook and Rich (1999)	3	591.56	0.42
33	C2-02	3	*589.10*	Cook and Rich (1999)	3	591.56	0.42
34	C2-03	3	*591.17*	Li and Lim (2002)	3	600.54	1.58
35	C2-04	3	*590.60*	Potvin and Bengio (1996)	3	610.01	3.29
36	C2-05	3	*588.88*	De Backer et al. (2000)	3	*588.88*	0.00
37	C2-06	3	*588.49*	Lau et al. (2003)	3	588.88	0.07
38	C2-07	3	*588.29*	Rochat and Tailard (1995)	3	589.58	0.22
39	C2-08	3	*588.32*	Rochat and Tailard (1995)	3	591.65	0.57
40	RC1-01	15	*1619.8*	Kohl et al. (1999)	16	1634.52	0.91
41	RC1-02	13	1530.86	Cordone and Calvo (2001)	15	*1492.89*	−2.48
42	RC1-03	11	*1261.67*	Shaw (1998)	13	1334.57	5.78
43	RC1-04	10	*1135.48*	Cordeau et al. (2001)	11	1215.62	7.06
44	RC1-05	13	1589.91	Tan et al. (2006)	15	*1546.43*	−2.73
45	RC1-06	12	*1371.69*	Tan et al. (2006)	14	1423.1	3.75
46	RC1-07	11	*1222.16*	Tan et al. (2006)	12	1300	6.37
47	RC1-08	10	*1133.90*	Tan et al. (2006)	12	1193.68	5.27
48	RC2-01	4	*1134.91*	Tan et al. (2006)	8	1308.76	15.32
49	RC2-02	4	*1130.53*	Tan et al. (2006)	8	1167	3.23
50	RC2-03	3	1026.61	Tan et al. (2006)	6	*1014.79*	−1.15
51	RC2-04	3	*798.41*	Mester et al. (2007)	4	881.88	10.45
52	RC2-05	4	1297.19	Mester et al. (2007)	7	*1210.68*	−6.67
53	RC2-06	3	*1112.20*	Yu et al. (2009)	6	1112.38	0.02
54	RC2-07	3	*1040.67*	Yu et al. (2009)	7	1059.62	1.82
55	RC2-08	3	*828.14*	Ibaraki et al. (2001)	5	882.06	6.51

*NV* number of vehicles

The Modified ABC algorithm produces best results (lowest distance) for 8 datasets out of 56 datasets, which means that 14 % of the results obtained a lower distance than the best-known results achieved by several approaches proposed over the years. It also achieved equal or better results when compared to the best individually published results with respect to minimising the total travelled distance for the VRPTW. Moreover, the Modified ABC algorithm provides competitive results for 20 datasets with same number of vehicles as compared to the best-known results.

**Fig. 3:**
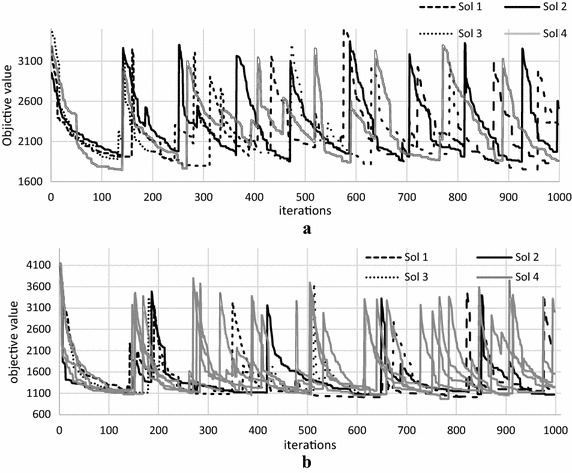
Behaviour of the abandoned solutions for ABC algorithm. **a** R1-01 dataset, **b** C1-03 dataset

**Fig. 4:**
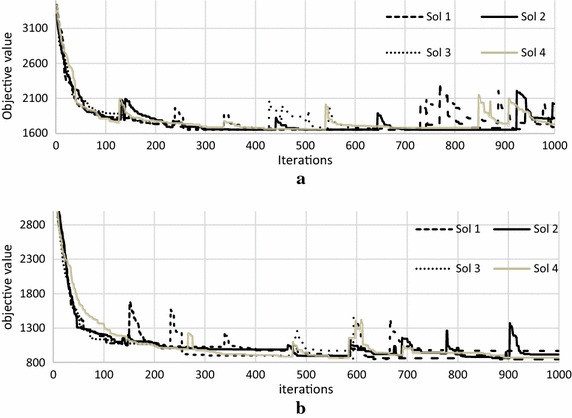
Behaviour of the abandoned solutions for Modified ABC algorithm. **a** R1-01 dataset, **b** C1-03 dataset

The effect of the proposed modification on ABC algorithm can be clearly observed in Figs. 3a, b and 4a, b. The lines represent the abandoned solutions, *x*-axis represents the iterations and the *y*-axis represents the objective value.

Figures 3 and 4 show the behaviour of the abandoned solutions on R1-01 and C1-03 datasets, respectively. It can be clearly seen in Fig. 3 that ABC algorithm offers *large moves* (the quality rose sharply) throughout the solution’s improvement process. This represents that the abandoned solution is replaced by a new solution where the objective value becomes high (as explained in modified ABC algorithm section), in which it looks like the search starts from the beginning, where the quality of the solution is almost similar to the earlier solution’s quality at the initial stage. Thus, it is hard for the ABC algorithm to further improve the solution again. Furthermore, at the end of the improvement process, these *large moves* are not accepted.

On the other hand, Fig. 4 shows the behaviour of the Modified ABC, where the figure demonstrates that when the abandoned solutions are replaced with new solution (the solution with random routes selected from the best solution). It can be seen that the quality of the solution goes slightly up (small moves), so the algorithm can easily improve the solution.

**Fig. 5:**
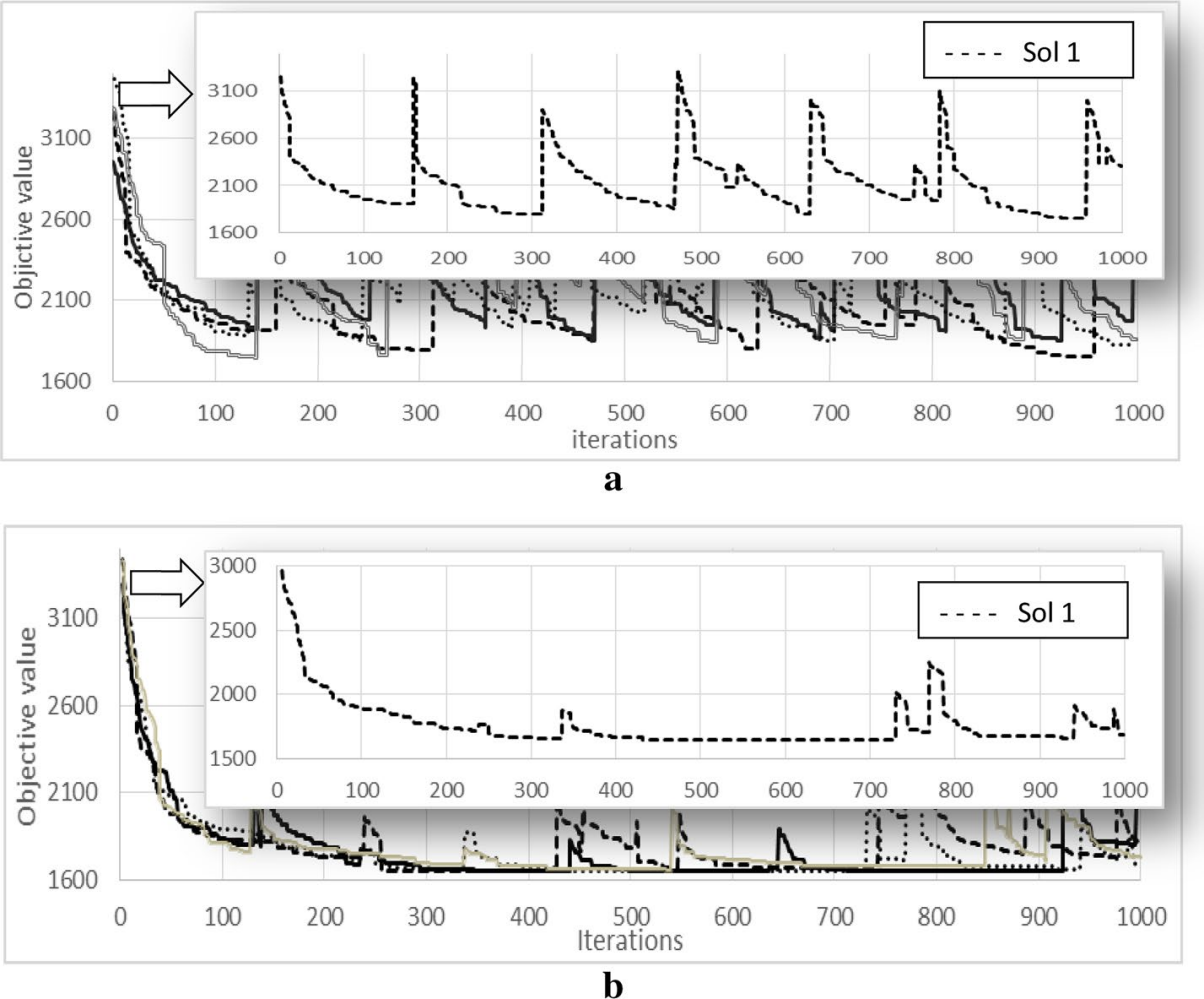
Behaviour of one solution from ABC and one solution form Modified ABC. **a** ABC on R1-01 dataset, **b** Modified ABC on R1-01 dataset

Figure 5 shows the behaviour of one solution extracted from the above figures for ABC and Modified ABC algorithms, in order to show the behaviour in details.

As shown in Fig. 5a the solution (Sol 1) becomes abandoned roughly at iterations (150, 300, 470, 550, 610, 770, 800, 970 and 990) and replaced by randomly generated solutions. The quality for the randomly generated solution is roughly 3100 (at iteration 150) which is most likely equal to the quality of the initial solution (before the ABC algorithm is performed). This shows that, due to the poor quality of the randomly generated solution to replace the abandoned solution, thus it is hard to be further improved by the ABC algorithm. However, in the Modified ABC, Fig. 5b the solution becomes abandoned roughly at iterations (250, 350, 750, 780, 950, and 980), and replaced by a new solution where the routes of the solution are selected from the best solution. From the figure, we can see that the quality of the replaced solution is roughly 1900 (at iteration 250) which is better compared to the quality of the initial solution (roughly 3000) before the replacement takes place. Thus, it helps the algorithm to easily improve the quality of the solution at every time the abandoned solutions are replaced.

## Conclusion

The Modified ABC algorithm proposed in this paper for the VRPTW appears to excel in the quality of its solution as well as in computational time. Experimental results show that the Modified ABC algorithm improves the results when using a memory by scout bees, where they can memorise the abandoned solutions and select one of these solution to be replaced by a new generated solution rather than replacing all the abandoned solutions by randomly generated solutions as in the original ABC algorithm. Overall comparison indicates that our Modified ABC algorithm is able to obtain the best results in comparison to state-of-the-art approaches, as represented by reducing the distance travelled, which is the main objective of the VRPTW.
